# Toward a self-wired active reconstruction of the hippocampal trisynaptic loop: DG-CA3

**DOI:** 10.3389/fncir.2013.00165

**Published:** 2013-10-21

**Authors:** Gregory J. Brewer, Michael D. Boehler, Stathis Leondopulos, Liangbin Pan, Sankaraleengam Alagapan, Thomas B. DeMarse, Bruce C. Wheeler

**Affiliations:** ^1^Department of Medical Microbiology, Immunology and Cell Biology, Southern Illinois University School of MedicineSpringfield, IL, USA; ^2^Department of Neurology, Southern Illinois University School of MedicineSpringfield, IL, USA; ^3^Pruitt Family Department of Biomedical Engineering, University of FloridaGainesville, FL, USA

**Keywords:** multielectrode array, dentate gyrus, GAD67, burst, GFAP

## Abstract

The mammalian hippocampus functions to encode and retrieve memories by transiently changing synaptic strengths, yet encoding in individual subregions for transmission between regions remains poorly understood. Toward the goal of better understanding the coding in the trisynaptic pathway from the dentate gyrus (DG) to the CA3 and CA1, we report a novel microfabricated device that divides a micro-electrode array into two compartments of separate hippocampal network subregions connected by axons that grow through 3 × 10 × 400 μm tunnels. Gene expression by qPCR demonstrated selective enrichment of separate DG, CA3, and CA1 subregions. Reconnection of DG to CA3 altered burst dynamics associated with marked enrichment of GAD67 in DG and GFAP in CA3. Surprisingly, DG axon spike propagation was preferentially unidirectional to the CA3 region at 0.5 m/s with little reverse transmission. Therefore, select hippocampal subregions intrinsically self-wire in anatomically appropriate patterns and maintain their distinct subregion phenotype without external inputs.

## Introduction

The mammalian hippocampus crucially encodes the formation of long-term episodic memories and spatial navigation, yet the staged encoding mechanisms remain elusive. While we know molecular details of many types of synapses in the major regions of the hippocampus important to learning and memory at the single neuron level, we don't know if these regions self-wire into the anatomically accurate network or require external electrical or chemical inputs. Further, brain functional studies are saddled with a tradeoff between high-spatial resolution (e.g., MRI, fMRI, EEG, EcoG) vs. high-temporal resolution (e.g., *in vivo* electrode arrays, single cell patch clamp). To bridge this gap, our strategy employs *in vitro* culture in an attempt to recapitulate entire *in vivo* brain regions in culture. Today a wide variety of cell types from various areas of the brain can easily be explanted, cultured *in vitro*, and studied in detail. While *in vitro* technology does provide exquisite temporal and spatial access it too has significant shortcomings. A key hurdle toward reconstructing brain areas *in vitro* has been the difficulty controlling the structural connectivity among cells to begin to recapitulate the *in vivo* architecture. The connections in the hippocampal formation of the brain uniquely propagate forward excitatory communication from one region to the next with the CA3 region distinctive for recurrent collateral excitation. We begin to create a functional tri-synaptic network of the hippocampal formation from the entorhinal cortex (EC) to the dentate gyrus (DG) to the CA3 to the CA1 (Cajal, 1968; Amaral and Lavenex, 2006). Other aspects of hippocampal anatomy not modeled here are the connections from the EC through the perforant path to the CA3 in a feed-forward fashion. In addition, the hippocampus receives modulatory inputs from the amygdala and basal forebrain. Output from the CA1 proceeds through the subiculum and returns to the EC to complete the loop. With smaller numbers of electrodes placed in the rat brain, others have monitored activity from each hippocampal region in behaving animals to describe specific patterns of activity for each region (Rolls and Kesner, 2006; Leutgeb et al., 2007), suggesting staged encoding, but we lack information about the inputs necessary to evoke these patterns and their network relationships.

**Figure 1:**
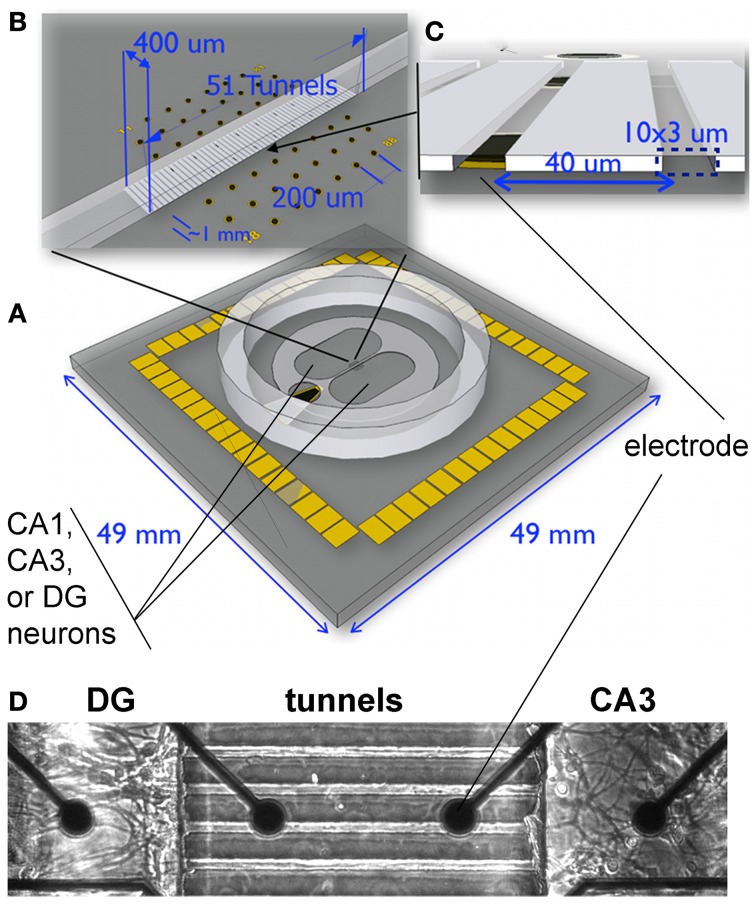
**Experimental system for reconstruction of hippocampal sub-regional circuits on multi-electrode array. (A)** Dual culture chamber on microelectrode array separated by microtunnels. Note ground electrode on lower left for common culture medium, **(B)** 51 tunnels of 400 um length aligned to the 8 columns of electrodes. **(C)** Fifty-one tunnels of 3 × 10 um cross section were separated by 40 um with alignment over one pair of dark electrodes shown. **(D)** Phase contrast imaging of live neurons shows how the tunnels promoted selective growth of axons from one compartment into another.

To achieve these staged connections, we combine microfabrication (MEMS) technology to channel connections between cultured subregions of the hippocampus on a multi-electrode array to simultaneously monitor activity (Figure 1). Inspired by Campenot (1977, 1987), the MEMS device creates compartments in which we separately place cells from each major area of the hippocampus (EC, DG, CA3, CA1) connected by micro-scale tunnels through which axons can pass between the wells to define neuronal communication pathways between each well (Taylor et al., 2005; Dworak and Wheeler, 2009; Pan et al., 2011; Kanagasabapathi et al., 2012). Due to the defined geometry of the hippocampus, the cells can be dissociated from micro-dissected subregions of DG, CA3, CA1, and EC (Mattson et al., 1989; Baranes et al., 1996; Zhao et al., 2001; Lein et al., 2004). Cells are loaded into the compartments after the device is placed over an array of extracellular electrodes (microelectrode array or MEA) to measure neural activity in each subnetwork as well as communication between the subnetworks through the tunnels (Morefield et al., 2000; Czarnecki et al., 2012; Downes et al., 2012; Kanagasabapathi et al., 2012; Dranias et al., 2013).

Here we reconstructed paired components of the tri-synaptic pathway, with a focus on the DG to CA3 connection to determine: (1) whether specific subregions of the hippocampus be reproducibly dissected as evidenced by region-restricted gene expression? (2) Will these regions maintain and establish their original identity in a uniform culture environment when removed from external hormonal gradients and input activity? (3) Given that CA3 development precedes DG *in vivo* (Bayer, 1980), is the natural axonal polarity of DG>CA3 intrinsically controlled by the neurons or does *in vivo* recapitulation of connectivity require external cues? (4) Do the dynamics of neural activity differentiate between each area *in vitro* and to what extent are they similar to activity patterns seen *in vivo*? We addressed the above issues by quantitative PCR of region-restricted gene expression, by evaluation of distinct spike and burst dynamics in each sub-region compartment and by establishing the polarity of directional communication between sub-regions, whether random or anatomically accurate from the DG to the CA3. Surprisingly, intrinsic capabilities of the DG neurons promote axon extension toward the CA3 neurons, with limited back propagation.

## Results

**Figure 2:**
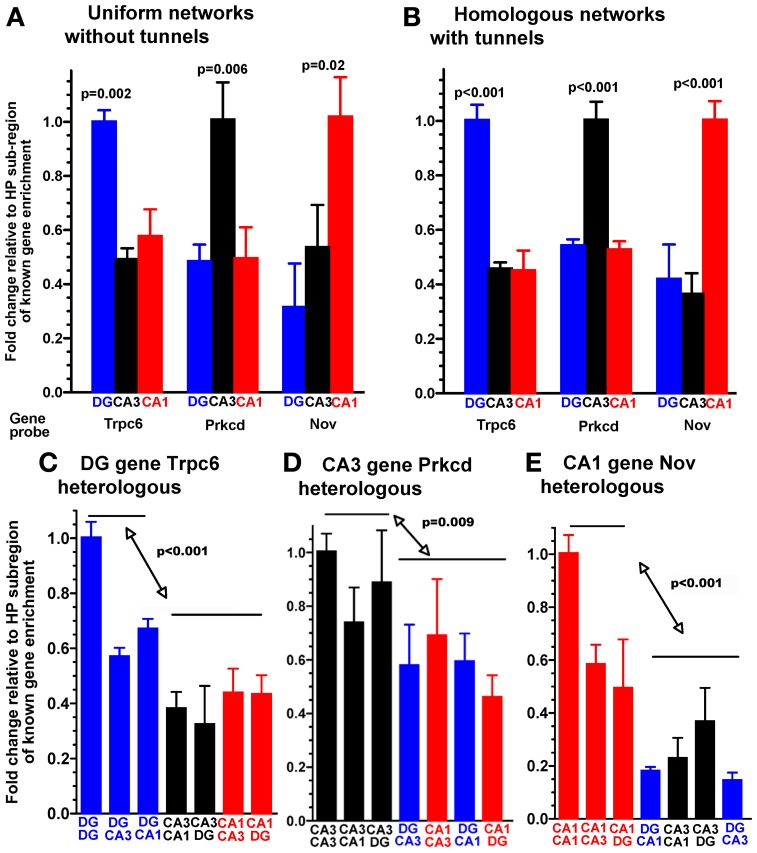
**Expression of region-enriched genes in sub-compartments phenocopies selective expression in the adult hippocampus as measured by qPCR**. Specific genes probed for expression after 3 weeks in **(A)** the indicated homogeneous random cultures without tunnel devices. Note enrichment of Trpc6 in the DG cultures, Prkcd in the CA3 cultures, and Nov in the CA1 cultures. **(B)** The same hippocampal subregions plated into each of two compartments of the tunnel device. Note same enrichment profile in the tunnel device as in the random cultures. **(C)** Enriched expression of the DG gene Trpc6 whenever DG neurons are present in heterologous combinations of hippocampal subregions cultured between tunnels, normalized to DG cultured on both sides of the tunnels. **(D)** Enriched expression of the CA3 gene Prkcd when CA3 neurons are present in heterologous combinations of hippocampal sub-regions cultured between tunnels, normalized to CA3 cultured on both sides of the tunnels. **(E)** Enriched expression of the CA1 gene Nov when CA1 neurons are present in heterologous combinations of hippocampal sub-regions cultured between tunnels, normalized to CA1 cultured on both sides of the tunnels. Note the similar expression of each region-specific gene to neurons of that region in combination with heterologous regions, while the other 4 combinations without this region express lower levels of this marker mRNA (*n* = 3 separate cultures).

In order to reconstruct subregions of the rat hippocampus, we microscopically dissected these regions from postnatal day 4 rats. At this time, the CA3 and CA1 are well-developed and the dentate granule and hilar region (DG) have nearly completed neurogenesis (Bayer, 1980). Single cell suspensions from each region were plated into separate compartments of a microfabricated PDMS device, positioned over a 60 electrode microarray (Figure 1). Cells were plated at physiological density ratios of 100 for DG to 33 for CA3 or 41 for CA1. Between the two compartments was a 400 um barrier perforated by a series of 51 narrow channels that excluded cell somata, but promoted growth of axons along the length of the tunnels (Taylor et al., 2005; Dworak and Wheeler, 2009; Berdichevsky et al., 2010; Pan et al., 2011; Kanagasabapathi et al., 2012; Wang et al., 2012). For quality control of the dissection and to determine whether the microdissected DG, CA1, and CA3 regions maintain their *in vivo* identity in culture, we performed qPCR on the neurons that developed in the compartments for 3 weeks. We selected several genes based on their demonstrated enrichment in specific regions of the adult hippocampus (Lein et al., 2004). When standard, uniform cultures from a single subregion were assessed from glass slips without tunnel devices, the adult animal enriched gene expression was replicated with detection of specific transcripts for each region DG, CA1, and CA3 cultured in the common culture medium (Figure 2A). But would the subregion types of expression be maintained across the microtunnel devices? Figure 2B shows that the same subregion-enriched gene expression is maintained between compartments with the same subregion in each compartment as well as in devices with different subregions on each side (Figures 2C–E). These results indicate the fidelity of the dissection and culture process as well as the ability of these hippocampal subregions to maintain their specific identities in the absence of external vascular, hormonal, or electrical instruction.

**Figure 3:**
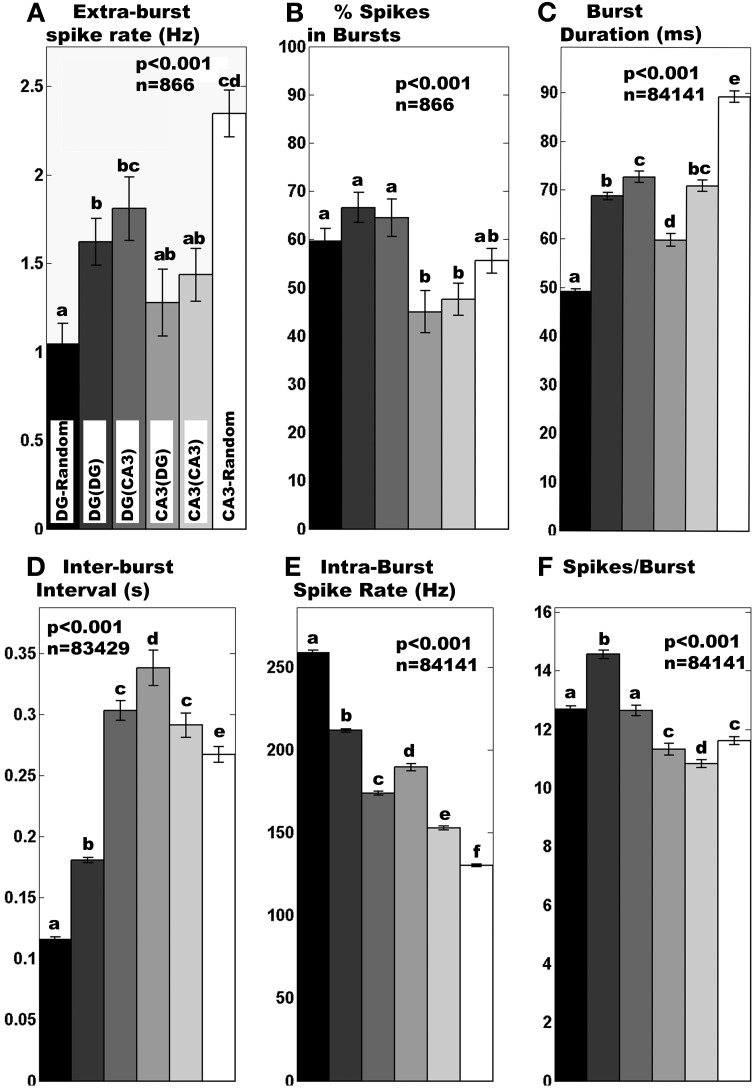
**Region-specific burst dynamics**. Normal distribution statistics indicate **(A)** mean extra-burst spike rate differs (i) in tunnels compared to corresponding random cultures and (ii) is higher for DG than CA3 apposed across tunnels. **(B)** Percent spikes in bursts are generally higher for any DG culture than any CA3 culture. Log normal distribution statistics apply to **(C–F)**. **(C)** Burst duration was longer for DG than CA3 when they were apposed. For random cultures without tunnel devices, DG burst durations are much lower than CA3 random cultures. **(D)** Inter-burst intervals are lengthened by 50% in DG apposed to CA3 compared to DG self-apposed across tunnels and 300% compared to DG in random networks. Similarly, inter-burst times are longer for CA3 apposed to DG than CA3 apposed to itself or random CA3 cultures. **(E)** Intra-burst spike rates are shortened by 20% in DG apposed to CA3 compared to DG self-apposed across tunnels but longer in the reverse direction. **(F)** Spikes per burst decreased by 14% in DG apposed to CA3 compared to DG self-apposed across tunnels and even less in the reverse direction. In all cases *n* displayed is total degrees of freedom from burst or non-burst segments from 3 min recordings of networks of 4 random DG, 4 random CA3, 8 DG(DG), 8 CA3(CA3), 5 DG(CA3), and 5 CA3(DG). Different letters above bars indicate significant differences (a shared letter indicates a non-significant comparison) by *post-hoc* Tukey multiple-comparison analysis after significant ANOVA, *p* < 0.05, normal distribution statistics.

We examined network spike and burst dynamics with the goal of decoding communication between hippocampal subregions. We recorded from paired compartments of DG and CA3 neurons as a model of this part of the brain anatomy. As controls, we recorded activity from networks comprised of either DG on both sides or CA3 neurons on both sides of tunnel-connected compartments or in random single compartment models. Regardless of configuration, 80% of electrodes were active with an average spike rate around 12 Hz in the NbAct4 medium (data not shown). However, burst dynamics differed between hippocampal subregions. The spike rate outside of bursts for DG increased with anatomically correct tunnel connection to CA3 neurons, while CA3 neurons showed the opposite trend (Figure 3A). Figure 3B shows that about 60% of spikes occurred within bursts in DG networks, regardless of configuration, while only 45% of spikes occurred in bursts in the CA3 networks apposed in tunnels. The average duration of each burst (Figure 3C) showed trends that mirrored the extra-burst spike rate, with DG apposed to CA3 showing longer burst durations while CA3 bursts were shorter. The larger changes in inter-burst interval (Figure 3D) showed a co-modulation upon anatomical connection with increased intervals for the DG and CA3 apposed configuration. Intraburst spike rate (Figure 3E) and spikes per burst (Figure 3F) also differed with configuration.

**Figure 4:**
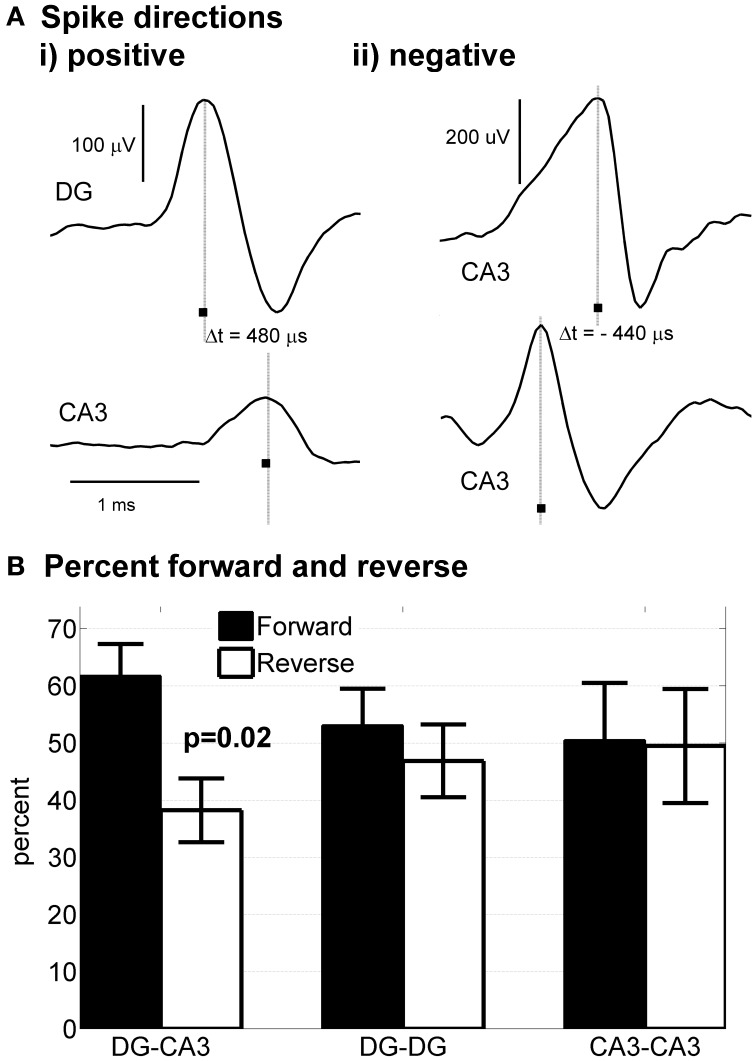
**Native polarity established from DG to CA3**. Delay times of spikes traveling in axons in tunnels were determined from the difference in spike times at two tunnel electrodes separated by 200 μm. **(Ai)** Example of spike travelling from DG to CA3 with a 480 μs delay indicating a velocity of 0.42 m/s. **(ii)** Example of spike propagation from the top to the bottom compartment (arbitrarily designated reverse direction for CA3-CA3). **(B)** Statistical analysis of directional propagation indicates 62% of tunnels spontaneously connect axons with anatomical accuracy from DG-CA3, while homologous regions across tunnels fail to show polarity (Wilcoxin non-parametric test).

*In vivo*, granule cells in DG uni-directionally synapse with pyramidal cells in CA3 with no back-propagation of connections from CA3 to DG. In our preparation, cells from DG and CA3 were plated simultaneously in apposing compartments that would permit connectivity in either direction. If axonal polarity of DG → CA3 is intrinsically controlled by the neurons (i.e., self-wire) in the absence of other external cues found *in vivo*, then polarity of connectivity from DG-CA3 should be maintained *in vitro* and could also account for the distinct burst dynamics of DG apposed to CA3. Previously, we showed that selective axon polarity from one side to the other, as opposed to bi-directional axon crossing, could be achieved in cortical neurons across tunnels by plating and culture of one side of a device followed after 1 week by plating and culture on the opposite side (Dworak et al., 2010; Pan et al., 2011). Here we tested whether intrinsic DG neuron properties would mimic *in vivo* conditions and preferentially cross the tunnels to innervate the CA3 neurons in the other compartment when both compartments were plated on the same day, with fewer axons crossing from CA3 to DG (reverse direction). This axon polarity could be measured in our devices by the direction of the time delay between spikes detected on the two microelectrodes embedded in the tunnels as shown in Figure 4Ai where a 0.48 ms delay from the DG side to the CA3 side over the 0.2 mm distance indicates a forward conduction velocity of 0.42 m/s. In contrast, when CA3 was connected to CA3 (or DG-DG), Figure 4Aii shows a 0.40 ms delay in the opposite direction, implying a reverse speed of 0.50 m/s that was evident in about half the spikes in these configurations. Individual tunnels were examined to determine whether axon conduction velocity was directionally polarized or whether evidence of multiple spike heights and shapes suggested several axons in a tunnel. Statistics based on all spike pairs above a high positive threshold indicated 81% unidirectional axon conduction from DG to CA3 at a mean velocity of 0.54 ± 0.02 m/s (SD., *n* = 9167 spike pairs). Other positive conduction velocities were DG:DG 0.47 ± 0.02 (*n* = 2615) and CA3:CA3 0.51 ± 0.01 (*n* = 1343) m/s, all within the range for the hippocampus *in vivo* (Patolsky et al., 2006).

Closer examination on a tunnel by tunnel basis revealed a more complicated situation. In 3 tunnels on one array, >99% of the spikes propagated from DG to CA3. In other tunnels, examination of the waveforms for negative directions from CA3 back to DG indicated multiple roughly simultaneous spikes, likely the spikes from two or more axons, whose sum was detected by the electrode as a shift in the peak within the 2 ms detection window. We never observed a tunnel with only back propagation, suggesting that these events are rare and supporting the conclusion that axons from DG neurons preferentially connect to CA3. For all the DG-CA3 tunnels with measurable spike pairs (*n* = 26 tunnels), more than >60% of the spikes propagated from the DG to CA3 direction (Figure 4B). This polarity contrasts with the nearly equal directional distributions of DG-DG and CA3-CA3.

**Figure 5:**
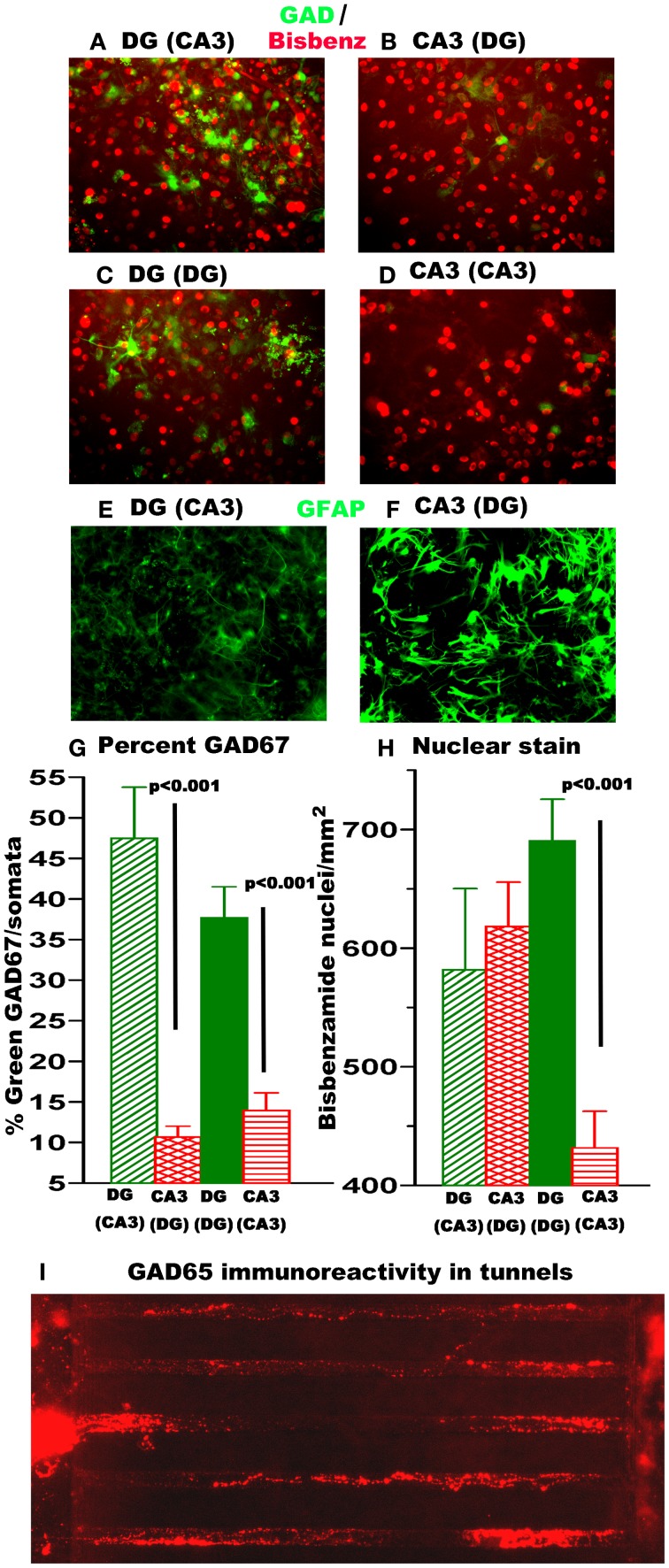
**DG networks contain 3–5× more GABAergic (GAD67-GFP) neurons than CA3 networks while CA3 has more astroglia**. Some GAD^+^ neurons traverse the tunnels. **(A–D)** Green is GAD67-GFP expression 11 days after infection with Lenti-virus with GAD-67 promoter fused to GFP. Red is pseudocolored for blue bisbenzamide labeled nuclei. **(E,F)** GFAP immunostain in DG or CA3 compartments. **(G)** Nuclei per somata from bisbenzamide stain for DNA. Note red vertical striped CA3 apposed to DG is 50% higher than CA3 by itself. **(H)** Percent GAD67 labeled neurons per nuclei. (*N* = 6 20× fields from each of 2 networks). **(I)** GAD65 immunolabeled axons traverse tunnels.

Differences in GABAergic neuron and astroglia content could also affect burst dynamics. Transfection of networks with a Lenti virus carrying a GFP reporter driven by the inhibitory neuron GAD67 promoter was used to evaluate neurons expressing the GABA synthetic enzyme GAD67. Figure 5 shows higher GAD^+^ inhibitory neuron density in heterologous sub-region connections in DG compared to CA3 (Figures 5A,B) and higher GAD^+^ inhibitory neurons/nucleus in homologous sub-region connection in DG compared to CA3 (Figures 5C,D). Quantitation of GAD67 neurons relative to nuclei (Figure 5G) showed a 5-fold increase in GAD^+^ neuron density in DG over CA3 for heterologous and a 3-fold increase in the homologous configuration. The dissected DG region which includes the hilus contains a higher percentage of GAD67 expressing neurons than CA3 (Harvey and Boksa, 2011) that could contribute to a stronger inhibitory drive to enable a higher percentage of spikes in bursts (Figure 3B) and a longer burst duration (Figure 3C) for DG than CA3. The same mechanism could also contribute to the longer inter-burst intervals in DG apposed to CA3 (Figure 3D) and offer more opportunity for higher extra-burst spike rates. Some of these GAD67 neurons sent axons across the tunnels (not shown), better seen with GAD65 immunoreactivity (Figure 5I), as a feed-forward inhibitory component (Cabezas et al., 2012).

By increasing glutamate uptake, higher astroglial density could also affect burst dynamics (Boehler et al., 2007). A nuclear count of more than 2-fold above the plating density for CA3 apposed to DG (Figure 5H) suggested proliferation of astroglia. Figures 5E,F show that GFAP stain for astroglia are indeed more activated in the CA3 side than the DG side.

## Discussion

Here we reconstructed rat hippocampal sub-regions in pairs connected by axon-conducting tunnels to demonstrate intrinsic retention of *in vivo* behavior in the absence of external electrical and hormonal stimuli. The subregions maintained their physiological distinctions based on qPCR expression of subregion-enriched genes, distinct spike dynamics, GABAergic neurons, astroglia, and preferential wiring direction.

Our observations on spike and burst dynamics are consistent with a synchronously connected network; neither DG nor CA3 operates as an independent oscillating/bursting center. Isolated DG neurons in the network burst at a higher rate than CA3 neurons, suggestive of regions preprogrammed to drive faster bursting DG onto slower bursting CA3, but operating in constrained fashion with predominantly forward connectivity from DG to CA3, but sufficient recurrent GABA-ergic innervation in DG and astroglia (Boehler et al., 2007) in CA3 to modulate the dynamic behavior. Further, the higher extra burst spike rate, slightly longer burst duration and GABAergic neuron density in DG provide extra drive from DG to CA3, establishing the background readiness of CA3 to be receptive to other inputs for learning or information fusion.

A key hurdle toward reconstructing brain areas *in vitro* has been the difficulty controlling the structural connectivity among cells to reflect, or even begin to adequately recapitulate the *in vivo* architecture in an *in vitro* model. A variety of novel *in vitro* technologies address this difficult problem. These technologies have been targeted toward modifying the surface chemistry to provide guidance cues that promote preferential attachment and growth (Boehler et al., 2012), using microfluidics (Morin et al., 2006), or alternatively, to capitalize on the intrinsic neuronal property to follow topographical features in their environment such as pillars (Dowell-Mesfin et al., 2004), ridges (Curtis and Wilkinson, 1997), or gradients (Hattori et al., 2010). The tunnel approach used in this paper confirms the intrinsic ability of *ex vivo* neurons to reconnect in an *in vivo* order (Czarnecki et al., 2012; Downes et al., 2012; Kanagasabapathi et al., 2012; Dranias et al., 2013).

Our model is a greatly reduced analogue of the *in vivo* circuit due to its anatomical incompleteness as a two-dimensional network. It fails to include modulatory cholinergic, noradrenergic, seratonergic, or dopaminergic inputs. It certainly lacks hormonal fluctuations and efficient removal of waste metabolites. With extracellular electrodes, we only monitor the net effects of thousands of synapses as individual action potentials per neuron.

Advantages of this model include direct stimulation and monitoring of electrical activity on time scales of milliseconds to weeks, pharmacologic access and most importantly, the ability to monitor inputs, axon communication, and outputs of the hippocampal region. The hippocampus is well-known for its different levels of information processing, but the details of the coding remain elusive. Our model has the potential to decode the information from the easily monitored spiking dynamics between hippocampal subregions. This technology will enable determination of the network integration of stimulation-dependent plasticity and how subregion-specific information patterns are reliably transmitted but differentially processed within each hippocampal subregion.

## Materials and methods

### Microfabrication of two-compartment tunnel devices

A multilayered mold made of photoresist SU-8 was fabricated on a silicon wafer. The first layer of the mold was made for the microtunnel structure. Briefly SU-8 2002 (Microchem, Inc.) was spun on a 4-inch silicon wafer at a nominal thickness of 3 μm, baked, exposed with the first mask, baked again, and developed. The second thicker layer of the mold was made for the well-structure. SU-8 2050 was spun on at a nominal thickness of 120 μm and then baked. The second mask was aligned to marks on the silicon wafer and then the second SU-8 film was exposed, baked again and developed. This mold was slowly filled with PDMS silicone rubber [polydimethylsiloxane; Sylgard 184 (Dow-Corning, Midland, Michigan) 10:1 ratio of pre-polymer (base)/cross-linker (curing agent)]. Once the PDMS spread over the entire wafer, it was heated for 2 h at 70°C for curing or in later devices, 10 h at 70°C. Two wells for culture and another smaller circular well for a reference electrode were formed on the peeled PDMS with a punch. Finally, a circular PDMS ring was placed around the entire device to form a chamber for holding cell culture media.

### Dissection of rat hippocampal subregions

The SIUSM LACUC approved these experiments as conforming to the Laboratory and Animal Use Guidelines of the NIH. To obtain neurons for electrical and genetic analysis, hippocampal sub-regions were isolated from anesthetized 4-day-old Sprague–Dawley rat pups as described (Mattson et al., 1989; Baranes et al., 1996; Zhao et al., 2001; Lein et al., 2004). The entire hippocampus was dissected away from the overlying neocortex of each brain hemisphere and removed as an intact structure for further sub-region dissection. The boundaries of the DG could be seen in the dissected hippocampus. Briefly, the CA1 or top portion of Ammon's horn was isolated at the natural division of the hippocampal fissure separating CA1 and DG-CA3. Using DG rostral and ventral ends as anchors, cuts were first made along the DG-CA1 boundary until the CA1 was separated and isolated. The CA3 sub-region (bottom remainder of Ammon's horn) was then dissected away from the DG following the clearly visible boundaries.

### Neuron culture

Hippocampal sub-region cells were plated at 1000 cells/mm^2^ for DG, 330 cells/mm^2^ for CA3, and 410 cells/mm^2^ for CA1 on poly-D lysine coated MEAs or glass cover-slips with attached PDMS micro-tunnels in NbActiv4™ medium (Brewer et al., 2008) (BrainBits, Springfield, IL). Poly-D-lysine (Sigma SLBB8061V) was dissolved at 37°C for 1 h in sterile water before application to the devices at 100 μg/mL and incubation overnight at room temperature. The PDMS tunnels served to connect axons from the separated source sub-region to the target sub-region. Sub-region cultures were plated at a ratio respective to their anatomical density *in vivo* (final ratios DG-CA3 3:1, CA3-CA1 1:1.25) (Braitenberg, 1981). Figure 1 depicts tunnel dimensions in relation to MEA dimensions. Briefly, the dimensions of 51 tunnels were 400 μm long, 10 μm wide, and 3 μm height with spacing 40 μm apart allowing for the coverage of seven electrode pairs in the middle of the MEA. Twenty-two electrodes were left uncovered in each of the top (target) and bottom (source) wells of the MEA (well area = 6.28 mm^2^). Homologous cultures connected with tunnels were plated at a 1:1 ratio. Homologous random cultures were plated on 15 mm glass slips (Assistant Brand, Carolina Biologicals). Source cultures were plated first on the bottom half of the MEA and incubated for 15–30 min before adding target cultures. Cultures were incubated at 37°C, 5% CO_2_, 9% O_2_ and saturating humidity (Thermo-Forma, Columbus, OH). Every 4–5 days, one-half of the culture medium was removed and replaced with the same volume of fresh medium up until the day of recording.

### Hippocampal sub-region RNA extraction and detection through qPCR

RNA was extracted from 3 week old cultures using 20 μL (device compartments) or 100 μL (random) Trizol (Life Technologies #15596-026) applied directly to glass cover-slips or tunnel wells. After addition of 20% volume chloroform and 5% glycogen (final 250 ug/mL), samples were centrifuged and precipitated according to the manufacturer. Five hundred nanograms of RNA was used to create a cDNA pool with the High Capacity RNA-to-cDNA Kit (Applied Biosystems #4387406) per instructions. Hundred nanograms of cDNA was used in a 20 μL multiplex Taqman reaction using primers known to be enriched in specific hippocampal sub-regions (Lein et al., 2004): Transient receptor potential cation channel 6 enriched in DG (Trpc6, Applied BioSystems 00677559); Protein kinase C delta enriched in CA3 (Prkcd, Applied BioSystems #00440891), family of serine and threonine specific protein kinases activated by calcium and secondary messenger diacylglycerol; Nephroblastoma overexpressed gene enriched in CA1 (Nov, Applied BioSystems #00578390), family of CCN secreted extracellular matrix associated signaling proteins; and polymerase (RNA) II (DNA directed) polypeptide A (POLR2a, Applied BioSystems #4448489) as the internal standard reference. POLR2a was chosen as the internal standard reference over the more conventional housekeeping gene GAPDH because the lower level of expression (Alan Brain Atlas) is more appropriate for other low expression genes. qPCR reactions were run with a 2× master mix (Applied Biosystems #4369016) in a StepOne Plus PCR system (Applied Biosystems) at the manufacturer's recommended optimized conditions of 10 min at 95°C for enzyme activation followed by 40 cycles of (15 s denaturation at 95°C and 1 min anneal/extend at 60°C). Primer expression was normalized to POLR2A with fold change differences determined using the 2^ΔΔCt^ method. Graphed results show fold changes in the hippocampal sub-region gene probe expression relative to the hippocampal sub-region of known gene enrichment.

### Multi-electrode arrays and recording

MEA's from Multichannel Systems (MCS, Reutlingen, Germany) consisted of 60 TiN_3_ electrodes with diameters of 30 μm and spacing of 200 μm, one of which served as ground. The spontaneous activity on the MEA's was measured using an MCS 1100× amplifier at 25 kHz sampling with a hardware filter of 1–3000 Hz at 37°C under continuous flow of hydrated, sterile 5% CO_2_, 9% O_2_, balance N_2_ (custom made, AGA, Springfield, IL). A Teflon membrane (ALA Scientific, Westbury, NY) was used to reduce evaporation and chances of contamination. MCRack software was used to record 3 week old cultures for 3 min of spontaneous activity.

### Spike activity analysis

Offline data analysis was performed using a modification of SpyCode V2.0 software (Bologna et al., 2010) including custom MATLAB scripts (The Mathworks, Natick, MA). After filtering the data with at 300 Hz high pass, spikes were identified as peak-to-peak amplitudes that exceeded 9 times the minimum root-mean-square of 200 ms contiguous windows. A dead-time or refractory period of 1 ms was assumed after each detected spike. Bursts were defined as 4 or more spikes with no greater than a 50 ms inter-spike interval.

Delay times of spikes within tunnels were used to determine directionality. Due to larger amplitudes in tunnels, thresholds were determined on a per-electrode basis as visually selected asymptotic minima of the continuous voltage distribution. Furthermore, the time stamp of any *spike event* was chosen as the time of occurrence of the voltage maximum within any such event. A histogram of delay times between spike pairs was constructed within the limits of ±280–600 μs, conforming to the known range of axonal conduction delays (Patolsky et al., 2006) and limited in resolution by the 40 μs sampling period. The histograms showed distinct peaks, each indicating the high precision in delay time that is consistent with an action potential propagating on a single axon past a pair of electrodes.

### GAD67 GFP reporter for GABAergic neurons

At 7 DIV, a fluorescent GAD67 lenti-virus reporter (System Biosciences #SR10023VA-1, 10 μg/ml) diluted in 8 μg/ml protamine sulfate for better adsorption (Sigma# P4020) was added to DG and CA3 sub-cultures with PDMS micro-tunnels on glass cover-slips. Cultures were incubated in NbActiv4 medium for two more weeks before imaging. At 3 weeks, cultures were switched from NbActiv4 medium to Hibernate Low Fluorescence—Glucose (BrainBits #112012) and bisbenzamide was added to stain cell nuclei for 2 min (final concentration 300 ng/ml, diluted in PBS, Sigma #B2261). Cultures were rinsed two times in Hibernate LF—glucose before being imaged. Images were taken through an Olympus 20×/0.45 objective, and recorded with a Retiga Exi CCD camera (QImaging, Surrey, BC, Canada). Image Pro^+^ software was used in digital analysis and display of the immunostain and nuclear stain. After flattening backgrounds, a constant density segmentation threshold was set for cell counts.

### GFAP or GAD65 immunostains for astroglia or GABAergic neurons

For immunostains, DG and CA3 sub-cultures with PDMS micro-tunnels were plated as described previously on glass cover-slips and cultured for 3 weeks, then fixed in 4% paraformaldehyde for 10 min. Cells were permeabilized and weakly antigenic sites blocked in 5% normal goat serum and 0.5% Triton X-100 in PBS. Conjugate mouse anti-GFAP/Alexa Fluor 488 (Molecular Probes #A21294) was diluted 1:500 in 5% NGS and 0.05 TX-100. Cells for incubated for 90 min at 22°C, then rinsed four times in PBS. Other antibodies were mouse anti-GAD65 1:250 (Sigma #G1166) with secondary Alexafluor 588 conjugated goat anti-mouse 1:1000 (Molecular Probes #11031). Nuclei of cells were stained for 2 min with bisbenzamide (final concentration 300 ng/ml, diluted in PBS, Sigma #B2261). Slips were rinsed two final times, imaged through an Olympus 20×/0.45 objective, and recorded with a Retiga Exi CCD camera (QImaging, Surrey, BC, Canada).

### Statistics

Statistical differences were determined by Student's *t*-test with *p* < 0.05 considered significant for two-way comparisons of data normally distributed. Log-normal adjustments were made when appropriate. *Post-hoc* Tukey adjustments for multiple-comparisons are reported after significant ANOVA. Statistical differences of spike times were determined by the Wilcoxon test with significance at *p* < 0.05 (Hollander and Wolfe, 1999).

### Conflict of interest statement

The authors declare that the research was conducted in the absence of any commercial or financial relationships that could be construed as a potential conflict of interest, with the exception that the culture medium, NbActiv4, was provided by BrainBits LLC, which is owned by one of the authors (Gregory J. Brewer).
